# MicroRNA-18a regulates the metastatic properties of oral squamous cell carcinoma cells via HIF-1α expression

**DOI:** 10.1186/s12903-022-02425-6

**Published:** 2022-09-05

**Authors:** Shihyun Kim, Suyeon Park, Ji-Hyeon Oh, Sang Shin Lee, Yoon Lee, Jongho Choi

**Affiliations:** 1 Department of Oral Pathology, College of Dentistry, Gangneung-Wonju National University, 7 Jukheon-gil, Gangneung-si, Gangwon-do Republic of Korea; 2 Department of Oral and Maxillofacial Surgery, College of Dentistry, Gangneung-Wonju National University, Gangneung, Republic of Korea; 3 Department of Conservative Dentistry, College of Dentistry, Gangneung-Wonju National University, Gangneung, Republic of Korea

**Keywords:** Oral squamous cell carcinoma, MicroRNA, Hypoxia, HIF-1α, Migration, Invasion

## Abstract

**Background:**

Rapid metastasis of oral squamous cell carcinoma (OSCC) is associated with a poor prognosis and a high mortality rate. However, the molecular mechanisms underlying OSCC metastasis have not been fully elucidated. Although deregulated expression of microRNA (miRNA) has a crucial role in malignant cancer progression, the biological function of miRNA in OSCC progression remains unclear. This study aimed to investigate the function of miRNA-18a in OSCC metastatic regulation via hypoxia-inducible factor 1α (HIF-1α).

**Methods:**

miRNA-18a-5p (miRNA-18a) expressions in patients with OSCC (n = 39) and in OSCC cell lines (e.g., YD-10B and HSC-2 cells) were analyzed using quantitative real-time polymerase chain reaction. HIF-1α protein expressions in OSCC cells treated with miRNA-18a mimics or combined with cobalt chloride were analyzed using western blotting. The miRNA-18a expression-dependent proliferation and invasion abilities of OSCC cells were analyzed using MTT assay, EdU assay, and a Transwell® insert system.

**Results:**

miRNA-18a expression was significantly lower in OSCC tissue than in the adjacent normal tissue. In OSCC cell lines, HIF-1α expression was significantly decreased by miRNA-18a mimic treatment. Furthermore, the migration and invasion abilities of OSCC cells were significantly decreased by miRNA-18a mimics and significantly increased by the overexpression of HIF-1α under hypoxic conditions relative to those abilities in cells treated only with miRNA-18a mimics.

**Conclusions:**

miRNA-18a negatively affects HIF-1α expression and inhibits the metastasis of OSCC, thereby suggesting its potential as a therapeutic target for antimetastatic strategies in OSCC.

**Supplementary Information:**

The online version contains supplementary material available at 10.1186/s12903-022-02425-6.

## Background

Oral squamous cell carcinoma (OSCC), the most common type of head and neck cancer worldwide, affects multiple sites, including the oral cavity, larynx, pharynx, and neck [1]. Approximately, 65,000 new OSCC cases are estimated to occur annually in the United States. [2]. In South Korea, 3310 patients were newly diagnosed with OSCC and died from the disease in 2015 [3]. Late diagnosis strongly influences the high mortality rate in patients with OSCC [4]. The presence of lymph node metastasis is one of the critical factors that determine the prognosis in such patients [5, 6]. Metastasis is associated with the migration and invasion of cancer cells from the primary tumor and is regulated in multiple steps and by various underlying molecular mechanisms. In OSCC, metastasis occurs predominantly in the cervical lymph nodes, which is an important determinant in OSCC diagnosis and patient survival [7, 8]. Despite the advancements in the detection and elimination of lymph node metastasis, this strategy has its own limitations, and the molecular mechanism underlying OSCC metastasis remains unclear.

MicroRNAs (miRNAs) are single-stranded noncoding RNAs composed of 21–25 nucleotides that negatively regulate gene expression in post-transcriptional processes by regulating messenger RNA (mRNA) degradation or translational suppression [9–11]. In OSCC, miRNAs have been reported to play a crucial role in the progression, metastasis, and the low long-term survival rates of the patients [9, 12]. Particularly, the miRNA-17-92 cluster, including miRNA-17, -18a, -19a/b, -20a, and -92a, has been reported to function in tumorigenesis and metastasis in several cancers, including thyroid, colon, lung, and renal cell carcinomas [13–16]. In this cluster, miRNA-17 is highly expressed and is related to recurrence in patients with OSCC according to its participation in cell cycle distribution [17]. Chang et al. detected the regulation of miRNA-17 and -20a in OSCC cells, which promoted the migration ability of OSCC cells. Clinically, the expression of these miRNAs is negatively associated with tumor node metastasis stage and lymphatic metastasis via direct binding to integrin β8 [18]. However, the expression level of this cluster in OSCC has not been verified, and it is unclear which miRNA in this cluster is involved in OSCC metastasis.

Hypoxia, an insufficient oxygen level in whole organisms or tissues, is a well-known critical factor in tumor microenvironments and pathophysiology. Hypoxia in tissues triggers various cellular molecular responses, including rapid cell division, metabolism, and abnormal blood vessel formation [19, 20]. Hypoxia also induces tumor progression and is one of the causes for chemotherapy and radiotherapy resistance in tumors [21]. Under hypoxic conditions, a family of transcription factors known as hypoxia-inducible factors (HIFs) is activated and plays a critical role in orchestrating multicellular signaling pathways by changing the expressions of hundreds of genes [22]. HIFs are heterodimeric transcription factors composed of HIF-1α and constitutively expressed HIF-1β. In particular, HIF-1α is a key molecule induced under hypoxic conditions during excessive tumor growth and can control the expression of target genes involved in tumor angiogenesis, proliferation, invasion, and metastasis in various cancers [23–26]. HIF-1α expression can regulate a panel of miRNAs, and certain miRNAs are able to target the protein expression of HIF-1α [27]. Wu et al. showed that hypoxia affected the induction level of type 1 collagen prolyl-4-hydroxylase, which led to the accumulation of Ago2 via an HIF-1α-dependent pathway [28]. In addition, Kelly et al. found that hypoxia-induced miRNA-210 expression suppressed the activity of glycerol-3-phosphate dehydrogenase 1-like, a regulator of HIF-1α, which resulted in an increase in HIF-1α stability and the expression of HIF-1α target genes [29]. However, other than the expression of HIF-1α, the precise molecular mechanisms underlying the clinical progression of OSCC are not well understood.

This study aimed to analyze the expression of HIF-1α and validate HIF-1α target miRNA candidates in OSCC. Furthermore, this study intended to determine if the interaction between HIF-1α and miRNAs regulates OSCC progression and metastasis.

## Methods

### Human tissues

In total, 39 paired normal and tumor tissue specimens were obtained from patients with OSCC. The biospecimens and data used in this study were obtained from the Biobank of Inje Paik University, Pusan National University and Keimyung University Dongsan Hospital (a member of the Korea Biobank Network), or Gangneung-Wonju Dental Hospital National University, Gangneung, South Korea according to the approved protocols. All studies received ethical approval from the Committees for Ethical Review of Research at Gangneung-Wonju National University (Institutional Review Board [IRB] No.: GWNUIRB-2020-26-1).

### Cell culture and transfection

The human OSCC cell lines YD-10B and HSC-2 were purchased from the Korean Cell Line Bank (Seoul, South Korea) and the Japanese Collection of Research Bioresources Cell Bank (Ibaraki, Osaka, Japan), respectively. These cell lines were cultured in Dulbecco’s modified Eagle’s medium with high glucose (Invitrogen, Carlsbad, CA, USA) supplemented with 1% penicillin/streptomycin and 10% fetal bovine serum (FBS; Gibco, Waltham, MA, USA). The cells were cultured in a humidified atmosphere at 37 °C with 5% CO_2_. To expose YD-10B and HSC-2 cells to hypoxic conditions, they were cultured in an HERA cell 150i incubator with 94% N_2_, 1% O_2_, and 5% CO_2_ at 37 °C. For the transfection of miRNA-18a, the cells were first cultured with reduced serum Opti-MEM (Gibco). After the cells reached 60% confluence, hsa-miRNA-negative control (25 nM; Bioneer, Daejeon, Korea) or hsa-miRNA-18a-5p (25 nM; Bioneer) was transfected into the cells using Lipofectamine RNAiMAX Transfection Reagent (Invitrogen). The cells were further cultured with 300 mM cobalt chloride (CoCl_2_) for 24 h after transfection. The mature sequence of hsa-miRNA-18a-5p used was as follows: 5′-UAA GGU GCA UCU AGU GCA GAU AG -3′.

### RNA extraction and quantitative real-time polymerase chain reaction (qRT-PCR) analysis

Total RNA was extracted using the TRIzol reagent (Invitrogen). For HIF-1α or miRNA-18a quantification, cDNA was synthesized using Superscript III Reverse Transcriptase (Invitrogen) or miRNA First-Strand Synthesis Kit (Takara Bio Inc., Shiga, Japan) according to the respective manufacturer’s instructions. The expression of HIF-1α or mature miRNAs was detected using a CFX96 Touch Real-Time PCR system (Bio-Rad Laboratories, Hercules, CA, USA) and SYBR Premix ExTaq (Takara Bio Inc., Shiga, Japan). The amplification conditions were as follows: initial denaturation at 95 °C for 10 s, followed by 40 cycles of denaturation at 95 °C for 5 s, and annealing and extension at 60 °C for 20 s. The primers used in the present study were as follows: forward-HIF-1α: 5′-CCA GTT ACG TTC CTT CGA TCA G-3′; reverse-HIF-1α: 5′-GTA GTG GTG GCA TTA GCA GTA G-3′; forward-hsa-miRNA-17: 5′-TGC TTA CAG TGC AGG TAG-3′; forward-hsa-miRNA-18a: 5′-AGG TGC ATC TAG TGC AG-3′; forward-hsa-miRNA-19a: 5′-GTT TTG CAT AGT TGC ACT A-3′; and forward-has-miRNA-20a: 5′-GTG CTT ATA GTG CAG GTA-3′. In addition, normalization was performed using the following primers: forward-GAPDH: 5′-CAA AGT TGT CAT GGA TGA CC-3′; reverse-GAPDH: 5′-CCA TGG AGA AGG CTG GGG-3′; and reverse-U6: 5′-AAA ATA TGG AAC GCT TCA CGA-3′. Relative expression levels were calculated using the 2^−ΔΔCt^ method. All experiments were performed in triplicate.

### Western blots

The cells were lysed with lysis buffer (RIPA buffer; Sigma), including Complete Protease Inhibitor Cocktail (Roche Diagnostics, Basel, Switzerland). The protein concentration of the lysates was calculated using the BCA Protein Assay Kit (Pierce, Rockford, IL, USA). Subsequently, 50 µg of the total lysate was mixed with 5 × loading dye (Bio-Rad) and separated using 8–12% sodium dodecyl sulfate polyacrylamide gel electrophoresis. After being electrophoresed, the proteins were transferred to a nitrocellulose membrane using a Wet/Tank Blotting System (Bio-Rad). The membranes were incubated with 5% bovine serum albumin (Sigma) in phosphate-buffered saline with 0.01% Tween-20 for 30 min at room temperature. Subsequently, they were incubated with rabbit monoclonal antibodies specific to anti-HIF-1α (1:1000 dilution; GeneTex, Inc., Irvine, CA, USA), GAPDH (1:3000 dilution; Ab Frontier, Seoul, Republic of Korea), and anti-mouse-Proliferating Cell Nuclear Antigen (PCNA) (1:1000 dilution; Cell Signaling Technology, Danvers, MA, USA) for 3 h at room temperature. Secondary antibodies were blocked using horseradish peroxidase (HRP)-conjugated goat anti-rabbit immunoglobulin G (IgG) (1:5000 dilution; Cell Signaling Technology) and HRP-conjugated goat anti-mouse IgG (1:10,000 dilution; Cell Signaling Technology). Specific bands were detected using an imaging system (VILBER Smart Imaging, Eberhardzell, Germany) and chemiluminescence reagent (Millipore Corporation, St. Louis, MO, USA). ImageJ image processing software (https://imagej.nih.gov/ij/) was used to analyze the intensity of each detected band, which was quantified as the fold change. All experiments were performed at least in duplicate.

### Cell proliferation assay

To analyze the proliferation ability of the OSCC cell lines, a 3-(4,5-dimethylyhiazol-2-yl)-2,5-diphenyl tetrazolium bromide (MTT) assay was performed to assess cell viability in proliferating cells and a Click-iT 5-ethynyl-2′-deoxyuurindine (EdU) assay was conducted to assess DNA synthesis in proliferating cells (each according to the manufacturer’s instructions). In the MTT assay, 3 × 10^3^ cells were seeded in a 96-well cell culture plate (Corning Inc., Corning, NY, USA) and cultivated with miRNA-18a or CoCl_2_ at 37 °C in an incubator with a humidified atmosphere containing 5% CO_2_. After 24 h, 200 µl of dimethyl sulfoxide (DMSO; Sigma) was added for 10 min, and the resulting formazan was dissolved in DMSO. The absorbance was then detected at 570 nm using a SpectraMax iD3 Multimode Microplate Reader (Molecular Devices, San Jose, CA, USA). In the EdU assay, 3 × 10^5^ cells were seeded on coverslips in cell culture dishes and cultured with miRNA-18a or CoCl_2_ at 37 °C in an incubator with a humidified atmosphere containing 5% CO_2_. The cells were maintained in EdU solution for 2 h, after which they were fixed using 4% paraformaldehyde for 20 min and permeabilized in 0.5% Triton X-100 for 15 min. Subsequently, the coverslips were stained using an EdU Cell Proliferation Kit, Alexa Fluor 488 (Abcam, Cambridge, UK) according to the manufacturer’s protocol. Each process was performed in a dark environment to protect the fluorescence from the light. The cells were imaged using fluorescence microscopy (Olympus, Tokyo, Japan), and the images were chosen randomly from seven parts of the coverslip. All experiments were performed at least in triplicate.

### Transwell migration and invasion assays

To analyze the migration and invasion abilities of the OSCC cells, a 24-well plate cell culture insert system (8.0-µm pore size; Corning, Inc.) was used. To determine the invasion abilities of the OSCC cells, the inserts were precoated with 5% collagen mixed with 40 µl of reduced serum Opti-MEM (Gibco). After transfection with the negative control or miRNA-18a, the reduced serum Opti-MEM medium was added to the upper insert and culture medium containing 10% FBS was added to the lower plate for 24 h. The inserts were then fixed with methanol and stained using Mayer’s Hematoxylin Histological Staining Reagent (Dako, Santa Clara, CA, USA) for 20 min at room temperature. The noninvaded cells in the upper chamber were scraped out using a cotton swap, and the number of invaded cells was counted using an inverted light microscope (Olympus, Tokyo, Japan) to make observations. The images of the migrated and invaded cells were randomly selected from seven parts of each insert, and the number of cells was calculated using ImageJ. All experiments were performed at least in triplicate.

### Statistical analysis

All results were presented as mean ± standard error of the mean. To confirm the normal distribution of the analysis data, Levene’s test was performed. If the data were normally distributed, student’s t-test was performed, and if they were not normally distributed, the Mann–Whitney test was performed. In this study, the differences were considered to be statistically significant for *p* values < 0.05.

## Results

### miRNA-18a and -20a target HIF-1α directly in patients with OSCC

**Fig. 1:**
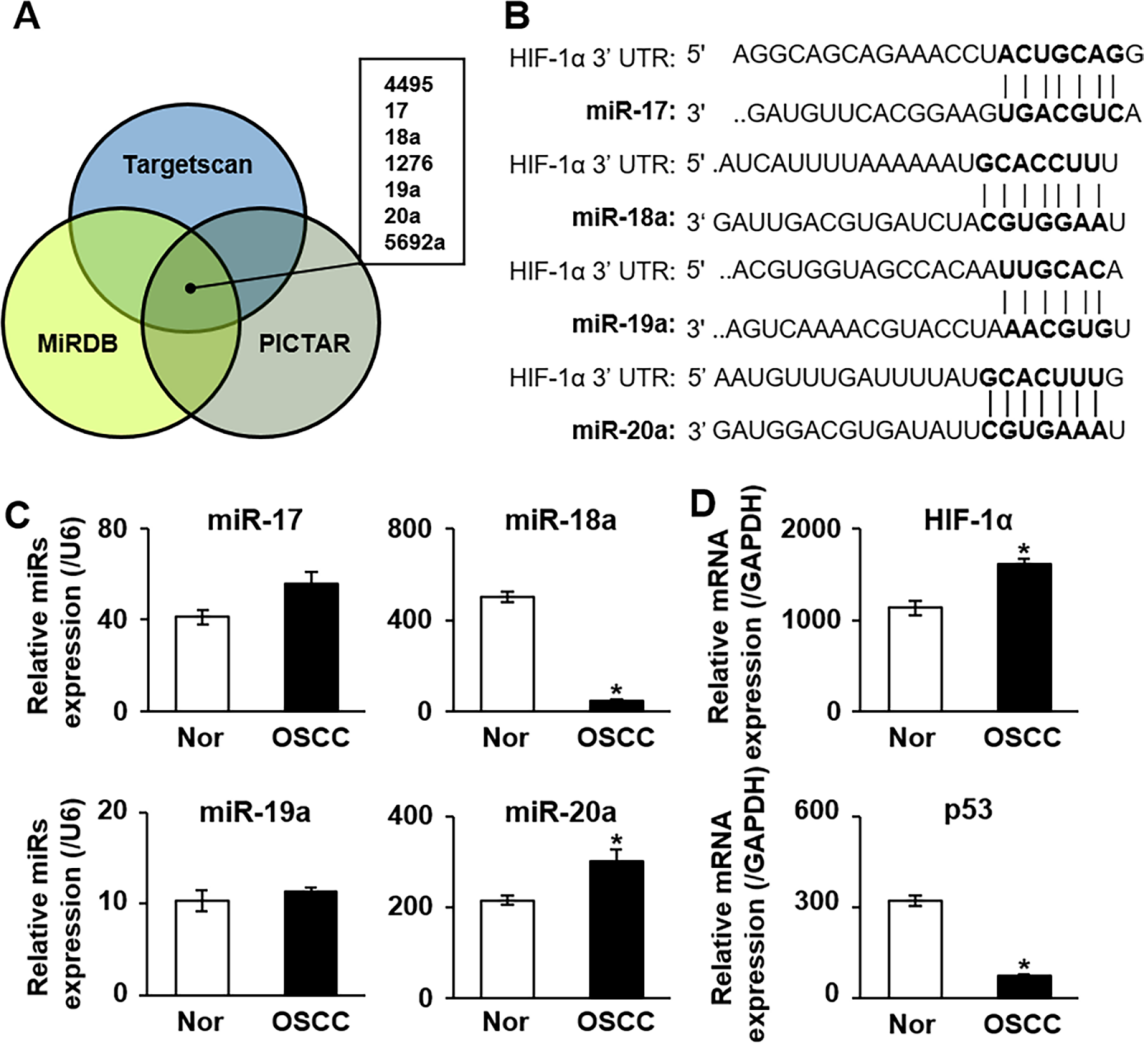
Identification of miRNA-18a associated with HIF-1α in OSCC. **A** Predictive analysis of HIF-1α and miRNAs according to three miRNA databases (Targetscan, MiRDB, and PICTAR). **B** Schematic drawing showing the specific binding sites between HIF-1α and the miR-17-92 cluster according to the results of a sequence alignment assay. **C** mRNA expression of miR-17, -18a, -19a, and -20a in OSCC relative to that in adjacent normal tissues, as assessed using qRT-PCR. *U6* was used as the control gene. **D** mRNA expression of HIF-1α and p53 in OSCC tissues relative to that in adjacent normal tissues according to qRT-PCR analysis. *GAPDH* was used as the control gene. *Significant difference in expression between the nontumor and tumor conditions (*p* < 0.05). Tissue samples were pooled. All experiments were performed at least in triplicate. miR-17, microRNA-17; miR-18a, microRNA-18a; miR-20, microRNA-20a; miRs, microRNAs; Nor, normal tissue; OSCC, oral squamous cell carcinoma

To determine miRNA candidates that might target HIF-1α in OSCC, we used three public algorithm program sets, namely, Targetscan, MiRDB, and PICTAR, to predict targets of miRNA. We found that miRNA-4495, -17, -18a, -1276, -19a, -20a, and -5692a potentially targeted HIF-1α (Fig. 1A). To verify these targets, we matched the miRNA sequences with the 3′-UTR region of HIF-1α mRNA. Sequence alignment showed that parts of miRNA-17, -18a, -19a, and -20a possessed complementary binding sites for the 3′-UTR of HIF-1α, whereas miRNA-4495, -1276, and -5692 did not (Fig. 1B). In clinical OSCC samples, the expressions of miRNA-18a and -20a were significantly decreased and increased, respectively, in the OSCC tissue relative to their expressions in the normal tissue (*p* < 0.05; Fig. 1C). Additionally, the expressions of HIF-1α and p53 (a tumor suppressor gene) were significantly increased and decreased, respectively, in the OSCC tissue relative to their expressions in the normal tissue (*p* < 0.05; Fig. 1D). Moreover, the expression of HIF-1α was significantly correlated with OSCC TNM staging including tumor size and lymph node metastasis  (*p* < 0.05; Additional file 1: Table S1). Collectively, these results suggest that miRNA-18a and -20a are direct targets of HIF-1α in OSCC although their expression patterns differed.

### Hypoxia suppresses the expression of miR-18a in OSCC cell lines

**Fig. 2:**
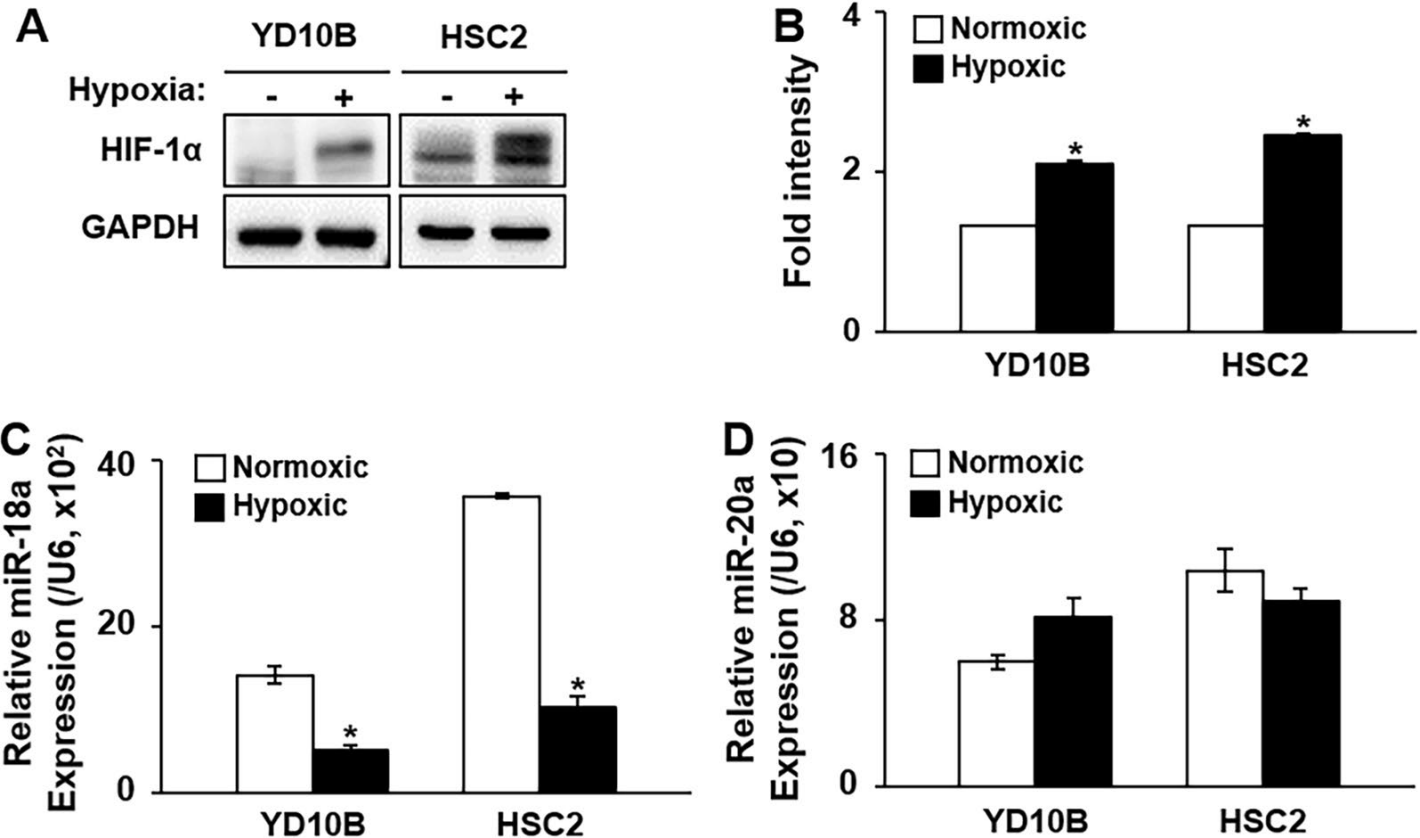
Expression of miR-18a in OSCC cell lines under hypoxic conditions. **A** Protein expression of HIF-1α in YD-10B and HSC-2 cells under hypoxic conditions according to western blot analysis. **B** Intensity graph showing the expression levels of proteins under hypoxic conditions in OSCC cell lines. *GAPDH* was used as the control gene. **C** mRNA expression of miR-18a and **D** miR-20a in OSCC cell lines under hypoxic conditions. *U6* was used as the control gene. *Significant difference in expression between the normoxic and hypoxic conditions (*p* < 0.05). miR-18a, microRNA-18a; miR-20, microRNA-20a; Normoxic, normoxic conditions; Hypoxic, hypoxic conditions. The original picture of western blots of **A** was shown in Additional file 2: Fig. S1

Some miRNAs are differentially expressed under hypoxia and are known as hypoxia-regulated miRNAs, which have been identified in several cancer types [30]. To determine if miRNA-18a and -20a are hypoxia-regulated miRNAs, we confirmed the expressions of HIF-1α and two miRNAs in YD-10B and HSC-2 cells cultured under normoxic or hypoxic conditions. The protein expression of HIF-1α was significantly increased in YD-10B and HSC-2 cells cultured under hypoxic conditions relative to its expression under normoxic conditions (*p* < 0.05; Fig. 2A, B). In contrast, the mRNA expression of miRNA-18a was significantly decreased in YD-10B and HSC-2 cells cultured under hypoxic conditions relative to its expression under normoxic conditions (*p* < 0.05; Fig. 2C). However, the mRNA expression of miRNA-20a was not altered in the two cell lines cultured under hypoxic conditions (Fig. 2D).

### miRNA-18a regulates the expression of HIF-1α in OSCC cell lines

**Fig. 3:**
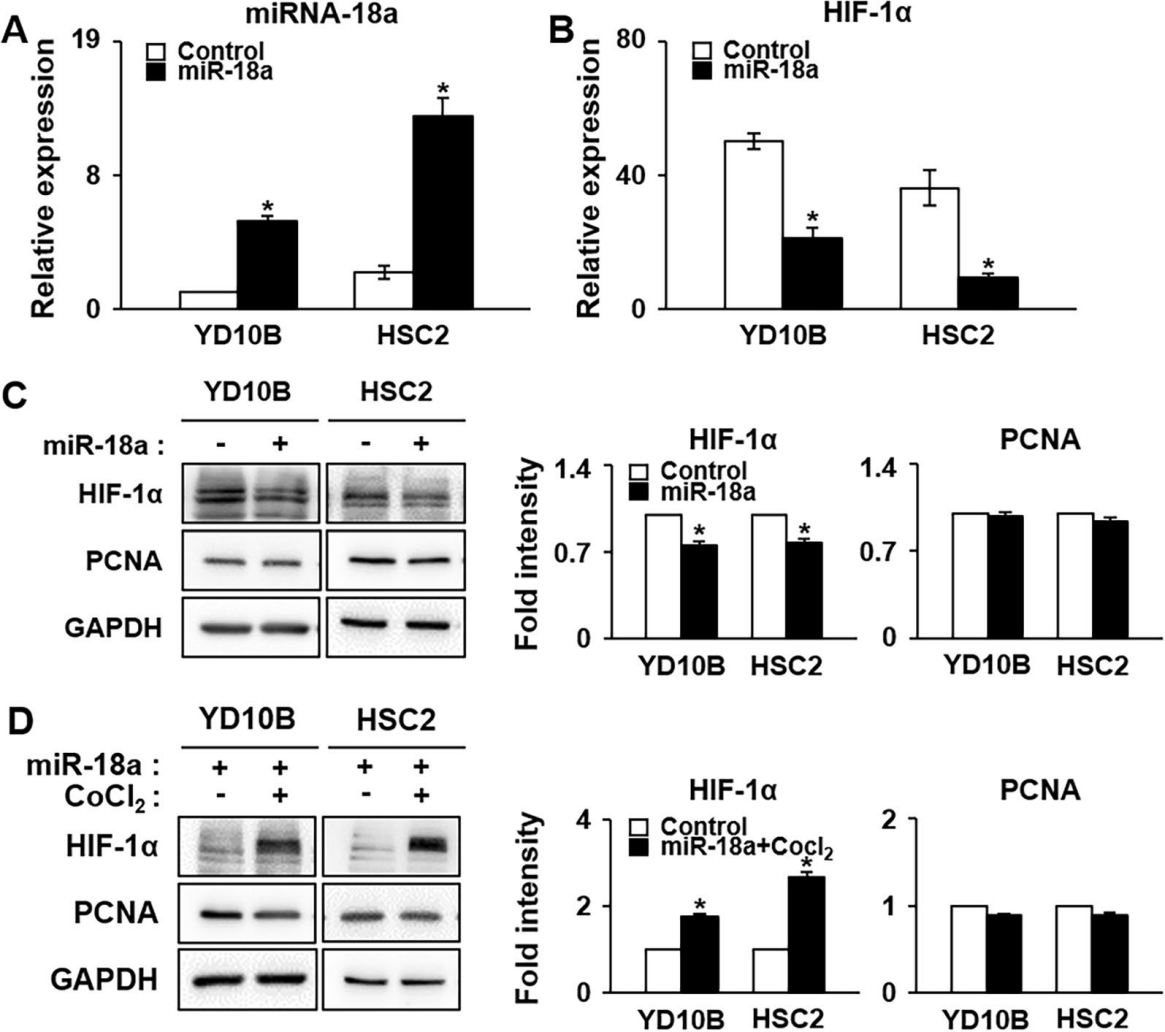
Expression of HIF-1α and PCNA induced by miRNA-18a in OSCC under hypoxic conditions. mRNA expression of **A** miRNA-18a and **B** HIF-1α induced by miRNA-18a mimics in OSCC cell lines according to qRT-PCR analysis. *U6* was used as the control gene. **C** Protein expression of HIF-1α and PCNA induced by **C** miR-18a mimics or **D** miRNA-18a and CoCl_2_ in OSCC cell lines according to western blot analysis. Intensity graph showing the expression level of proteins after treatment with **C** miR-18a mimics or **D** miRNA-18a and CoCl_2_ in OSCC cell lines, as determined with western blotting. *Significant difference in expression between control- and miRNA-18a-treated or miRNA-18a and CoCl_2_-treated OSCC cell lines (*p* < 0.05). *GAPDH* was used as the control gene. All experiments were performed at least in triplicate. miR-18a, microRNA-18a; CoCl_2_, cobalt chloride. The original pictures of western blots of **C** and **D** were shown in Additional file 2: Fig. S2, S3 and Fig. S4, S5.

To determine if miRNA-18a directly regulates the expression of HIF-1α in OSCC cell lines, we confirmed HIF-1α expression in YD-10B and HSC-2 cells treated with miRNA-18a mimics. miRNA scrambled control (control) or miRNA mimics were transfected into each OSCC cell line. The qPCR analysis showed an approximately five-fold increase in miRNA-18a expression in YD-10B and HSC-2 cells treated with miRNA-18a mimics compared with its expression in control cells (*p* < 0.05; Fig. 3A), which resulted in significantly decreased mRNA expression of HIF-1α relative to that in control cells (*p* < 0.05; Fig. 3B). Indeed, miRNA-mediated translation expression is usually coupled with the target degradation of mRNA [31]. Furthermore, we observed that the protein expression of HIF-1α was significantly decreased in YD-10B and HSC-2 cells treated with miRNA-18a mimics relative to its expression in control cells (*p* < 0.05; Fig. 3C). To test the re-expression of HIF-1α, we used a combination treatment of YD-10B and HSC-2 cells, i.e., treatment with CoCl_2_ after miRNA-18a mimic treatment. Accordingly, HIF-1α protein expression was significantly increased in YD-10B and HSC-2 cells treated with miRNA-18a mimics and CoCl_2_ relative to its expression in cells treated only with miRNA-18a mimics (*p* < 0.05; Fig. 3D). However, the miRNA-18a mimics and CoCl_2_ had no effect on the protein expression of PCNA (Fig. 3C, D). These results suggest that the expression of HIF-1α was regulated by miRNA-18a in YD-10B and HSC-2 cells.

### miRNA-18a inhibits the metastatic, but not proliferative, properties of OSCC cells

**Fig. 4:**
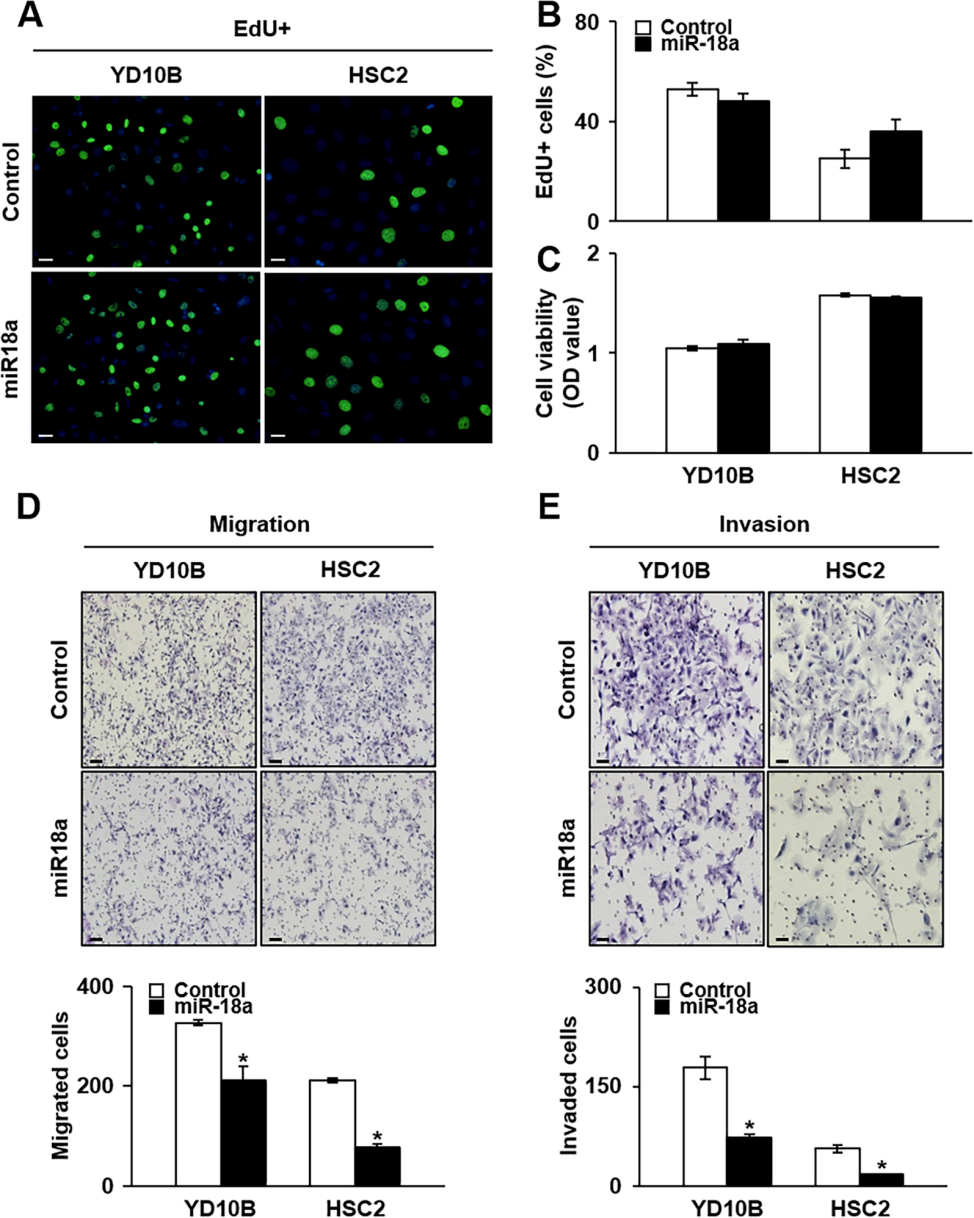
Cell viability, migration, and invasion abilities in OSCC cell lines according to miRNA-18a treatment. **A** Representative images acquired after an EdU assay in YD-10B and HSC-2 cells with and without miRNA-18a treatment (scale bar: 200 μm; original magnification: × 10). **B** Graphs showing the mean positive cell number for proliferation in five random regions in each well in the two tested OSCC cell lines. **C** Graphs showing cell viability, as measured using an MTT assay in YD-10B and HSC-2 cells. Representative images of **D** migration ability (scale bar: 200 μm; original magnification: × 10) and **E** invasion ability (scale bar: 200 μm; original magnification: × 20) in YD-10B and HSC-2 cells transfected with miRNA-18a. Graphs represent the mean migrated and invaded cells induced by miRNA-18a treatment in YD-10B and HSC-2 cells in five random regions in each well (bottom panels). *Significant difference between the control- and miRNA-18a-treated conditions (*p* < 0.05). miR-18a, microRNA-18a

Overexpression of miRNA-18a has previously been reported to promote the proliferation ability of OSCC cells in vitro [32]. To further examine the effect of miRNA-18a expression on the proliferation ability of OSCC cells, we performed EdU and MTT assays to analyze the proliferative abilities of YD-10B and HSC-2 cells treated with miRNA-18a mimics. Compared with the control cells, EdU incorporation into cells treated with miRNA-18a mimics had no significant effect (Fig. 4A, B). Consistently, there was no significant difference in the MTT absorbance of YD-10B and HSC-2 cells treated with miRNA-18a mimics relative to that of the control cells (Fig. 4C). Additionally, a Transwell migration assay performed to determine if miRNA-18a had an effect on the metastatic properties of OSCC cells showed that the number of migrated cells decreased significantly in miRNA-18a mimic-treated YD-10B and HSC-2 cells relative to the number in the control cells (*p* < 0.05; Fig. 4D) (Additional files 1 and 2). Moreover, the number of invaded cells decreased significantly in YD-10B and HSC-2 cells treated with miRNA-18a mimics (*p* < 0.05; Fig. 4E). Collectively, these findings indicate that miRNA-18a plays a role in the metastatic properties of OSCC.

### miRNA-18a regulates the metastatic properties of OSCC primarily via HIF-1α

**Fig. 5:**
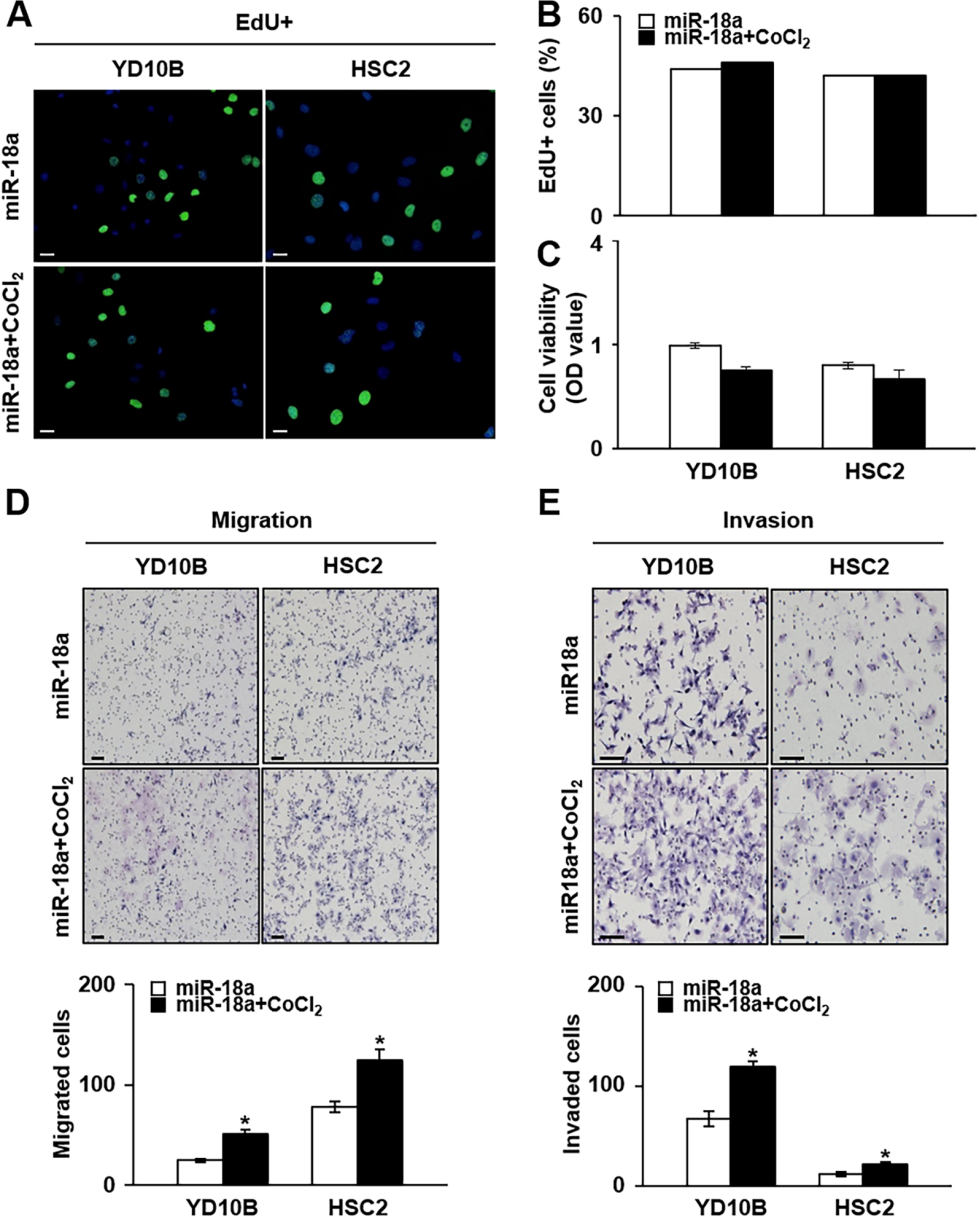
Cell viability, migration, and invasion abilities of OSCC cell lines under hypoxic conditions with miRNA-18a treatment. **A** Representative images from the EdU assay in which YD-10B and HSC-2 cells were treated with CoCl_2_ and miR-18a or miRNA-18a alone (scale bar: 200 μm; original magnification: × 10). **B** Graphs showing the mean positive cell number for proliferation in five random regions in each well in the two tested OSCC cell lines. **C** Graphs showing cell viability, as measured by an MTT assay in YD-10B and HSC-2 cells. Representative images of **D** migration ability (scale bar: 200 μm; original magnification: × 10) and **E** invasion ability (scale bar: 200 μm; original magnification: × 20) in YD-10B and HSC-2 cells under CoCl_2_ treatment induced with miRNA-18a treatment. Graphs showing the mean number of migrated and invaded cells induced by miRNA-18a treatment in YD-10B and HSC-2 cells under hypoxic conditions (bottom panels). These values were obtained from five random regions in each well. *Significant difference between cells treated with miRNA-18a alone and those treated with miRNA-18a under hypoxic conditions (*p* < 0.05). miR-18a, microRNA 18a; CoCl_2_, cobalt chloride

To determine if the proliferative properties induced by miRNA-18a were mediated by HIF-1α expression, we investigated the effects of miRNA-18a mimic treatment alone or in combination with CoCl_2_. The proliferation assay showed that EdU incorporation into YD-10B and HSC-2 cells treated with miRNA-18a mimics and CoCl_2_ was not significantly different from that in cells treated with miRNA-18a mimics alone (Fig. 5A, B). However, there was no significant difference in the MTT absorbances of YD-10B and HSC-2 cells treated with both miRNA-18a mimics and CoCl_2_ relative to those of cells treated with miRNA-18a alone (Fig. 5C). To determine if the metastatic properties induced by miRNA-18a were mediated by HIF-1α expression, we confirmed the migration and invasion ability of YD-10B and HSC-2 cells treated with miRNA-18a mimics alone or in combination with CoCl_2_. The number of migrated cells increased significantly in YD-10B and HSC-2 cells treated with both miRNA-18a mimics and CoCl_2_ relative to that in cells treated with miRNA-18a mimics alone (*p* < 0.05; Fig. 5D). Furthermore, the number of invaded cells increased significantly in YD-10B and HSC-2 cells treated with miRNA-18a mimics and CoCl_2_ in combination relative to that in cells treated only with miRNA-18a mimics (*p* < 0.05; Fig. 5E). These results allude that the miRNA-18a-induced metastatic properties of OSCC were mediated by HIF-1α expression although the results were inconsistent with the proliferative properties of OSCC cells.

## Discussion

Despite the advancements in diagnosis and treatment, the 5-year survival rate of patients with OSCC remains low; thus, the disease is an unresolved public health problem [33]. Several studies have highlighted the fundamental role of miRNAs in tumor progression [34, 35]. The miRNA-17-92 cluster is a well-known collection of miRNAs, the deregulation of which has been investigated in various tumor types [36, 37]. Many studies have highlighted the conflicting pro-oncogenic or antioncogenic roles of these miRNAs in different cancer types, and this disagreement probably reflects the polycistronic structure of the clusters [38, 39]. In this study, the expression of the miRNA-17-92 cluster, including miRNA-17, -18a, -19a, and -20a, was inconsistent in patients with OSCC. For example, miRNA-18a was rarely expressed in patients with OSCC, whereas miRNA-20a was highly expressed. Thus, we hypothesized that these two miRNAs have different functions: miRNA-18a acts as an antioncogene, whereas miRNA-20a acts as a pro-oncogene and participates in the progression and tumorigenesis of OSCC.

As one of the most multifunctional and conserved miRNAs, the altered expression of miRNA-18a has been detected in various pathological and physiological processes, including cell proliferation, apoptosis, cancer invasion, and metastasis [40]. Relative to the other members of this cluster, miRNA-18a is less well-studied, and our limited understanding of it comes primarily from expression studies in human cancer cells. For example, Liu et al. showed that miRNA-18a is upregulated in hepatocellular carcinoma cells and that its expression promotes the proliferation of these cells by blocking the translation of estrogen receptor alpha [41]. In addition, the inhibition of miRNA-18a is known to induce the proliferation of thyroid cancer cells, such as ARO and FRO cells, because of caspase cascade activation, which results in apoptosis [15]. Thus, miRNA-18a may be a potential therapeutic target for cancer therapy. However, the functional role of miRNA-18a in OSCC progression is yet to be confirmed [42]. Studies have shown that the expression is upregulated in hypopharyngeal and laryngeal squamous cell carcinomas (SCCs) but downregulated in oropharyngeal SCC and that this differential expression markedly affects human papillomavirus (HPV) status [43–45]. In the present study, to exclude the expression of HPV-status-dependent miRNAs, two types of OSCC cell lines, YD-10B and HSC-2 cells (i.e., HPV-negative cell lines [46–48]), were used to determine the effects of miRNA-18a in OSCC progression. Unexpectedly, we found that miRNA-18a expression was not associated with the viability of either OSCC cell line. Similarly, Liu et al. demonstrated that miRNA-18a had no effect on cell viability in cervical cancer or on apoptosis after transfection but did affect cell viability in cervical cancer after radiotherapy [49]. Thus, miRNA-18a apparently participates in cellular senescence by regulating DNA double-strand breaks rather than proliferation and apoptosis [50]. However, further investigation into the cellular responses to DNA double-strand breaks and cellular senescence in relation to miRNA-18a expression during OSCC progression is required.

Solid tumor cells commonly encounter low nutrient and oxygen conditions in their region of origin during tumor development and progression, and hypoxic conditions in solid tumors are known to promote the activation of HIF-1α [51]. Hypoxic conditions mediate tumor growth, metabolism, and metastasis via several mechanisms, including inactivation of tumor suppressor genes and activation of oncogenes via miRNAs [27] known as hypoxamiRNAs [52]. Using bioinformatics analysis, we found evidence showing that four candidate miRNAs among the miRNA-17-92 cluster target HIF-1α, and miRNA-18a was verified as a target of HIF-1α expression. Indeed, the expression of miRNA-18a was markedly altered in OSCC cell lines under hypoxic conditions; thus, we hypothesized that it has a role in the regulation of OSCC cell behavior. In addition, overexpression of miRNA-18a inhibited the migration and invasion ability of OSCC cells under hypoxic conditions. Based on the effects of miRNA-18a under hypoxic conditions and the association between miRNA-18a and HIF-1α, it is possible that miRNA-18a affects the migration and invasion abilities of OSCC cells via HIF-1α.

One limitation of this study is that the mechanism underlying the migration and invasion ability of OSCC cells that depended on the altered expression of miRNA-18 and/or HIF-1α under hypoxic conditions was not investigated in vivo. Therefore, further studies should be conducted to address this limitation.

## Conclusion

This study showed that miRNA-18a is downregulated in patients with OSCC and that it affects the migration and invasion of OSCC cells (YD-10B and HSC-2 cell lines) under hypoxic conditions. Furthermore, miRNA-18a was identified to be a target miRNA of HIF-1α. Collectively, these results indicate that miRNA-18a is a potential target for antimetastatic strategies in OSCC. However, the interaction between miRNA-18a and HIF-α and how it regulates the molecular mechanisms underlying metastasis should be further investigated to clarify the effect of miRNA-18a on OSCC metastasis.

## Supplementary Information

**Additional file 1. Supplementary table 1.** Characteristics of the studies of 39 patients with OSCC, and association between HIF-1α expression these variables.

**Additional file 2. Figure S1.** The original picture of western blots of Figs. 2A were shown in Figs. S1 (the red box indicated the representative picture in duplicate). Protein expression of HIF-1α in YD-10B and HSC-2 cells under hypoxic conditions by western blot analysis. All membranes were cut into small pieces and incubated with each antibodies. GAPDH was used as the control gene. Merge images indicate combination with bright field and chemiluminescence. The chemiluminescence images were time exposures of minimum (Low exposure) and maximum (High exposure) duration by the Fusion Solo Vilber Lourmat system. All images were unprocessed files.**Figure S2.** The original picture of western blots of Figs. 3C were shown in Figs. S2 (the red box indicated the representative picture in duplicate). Protein expression of HIF-1α and PCNA induced by miRNA-18a mimics in YD-10B cells by western blot analysis. All membranes were cut into small pieces and incubated with each antibodies. GAPDH was used as the control gene. Merge images indicate combination with bright field and chemiluminescence. The chemiluminescence images were time exposures of minimum (Low exposure) and maximum (High exposure) duration by the Fusion Solo Vilber Lourmat system. All images were unprocessed files. **Figure S3.** The original picture of western blots of Figs. 3C were shown in Figs. S3 (the red box indicated the representative picture in duplicate). Protein expression of HIF-1α and PCNA induced by miRNA-18a mimics in HSC-2 cells by western blot analysis. All membranes were cut into small pieces and incubated with each antibodies. GAPDH was used as the control gene. Merge images indicate combination with bright field and chemiluminescence. The chemiluminescence images were time exposures of minimum (Low exposure) and maximum (High exposure) duration by the Fusion Solo Vilber Lourmat system. All images were unprocessed files. **Figure S4.** The original picture of western blots of Figs. 3D were shown in Figs. S4 (the red box indicated the representative picture in duplicate). Protein expression of HIF-1α and PCNA induced by miRNA-18a mimics or miRNA-18a and CoCl2 in YD-10B cells by western blot analysis. All membranes were cut into small pieces and incubated with each antibodies. GAPDH was used as the control gene. Merge images indicate combination with bright field and chemiluminescence. The chemiluminescence images were time exposures of minimum (Low exposure) and maximum (High exposure) duration by the Fusion Solo Vilber Lourmat system. All images were unprocessed files. **Figure S5.** The original picture of western blots of Figs. 3D were shown in Figs. S5 (the red box indicated the representative picture in duplicate). Protein expression of HIF-1α and PCNA induced by miRNA-18a mimics or miRNA-18a and CoCl2 in HSC-2 cells by western blot analysis. All membranes were cut into small pieces and incubated with each antibodies. GAPDH was used as the control gene. Merge images indicate combination with bright field and chemiluminescence. The chemiluminescence images were time exposures of minimum (Low exposure) and maximum (High exposure) duration by the Fusion Solo Vilber Lourmat system. All images were unprocessed files.

## Data Availability

The data generated or analyzed during this study are included in the published article.

## Abbreviations

OSCC: Oral squamous cell carcinoma
miRNA: MicroRNA
HIF-1α: Hypoxia-inducible factor 1α
CoCl_2_: Cobalt chloride
PCNA: Proliferating cell nuclear antigen
