# Supplementary material for: MicroRNA-18a regulates the metastatic properties of oral squamous cell carcinoma cells via HIF-1α expression
Source: BMC Oral Health. 2022 Sep 5;22:378. doi: 10.1186/s12903-022-02425-6 (PMC9442921; doi:10.1186/s12903-022-02425-6)
Supplement: Supplementary file 1 — Additional file 1. Supplementary table 1. Characteristics of the studies of 39 patients with OSCC, and association between HIF-1α expression these variables. [file 12903_2022_2425_MOESM1_ESM.docx]

**Supplementary table 1.** Characteristics of the studies of 39 patients with OSCC, and association between HIF-1α expression these variables.

| **Clinical variables** | **NO. of patients** | **HIF-1α expression** | | **P** |
| --- | --- | --- | --- | --- |
|  |  | Low | High |  |
| Overall | 39 | 8 | 31 | ㅡ |
|  |  |  |  |  |
| Age |  |  |  | 0.592 |
| ≤ | 9 | 5 | 4 | ㅡ |
| > | 30 | 14 | 16 | ㅡ |
|  |  |  |  |  |
| Tumor location |  |  |  | 0.482 |
| Buccal | 11 | 6 | 5 | ㅡ |
| Tongue | 15 | 6 | 9 | ㅡ |
| Gingival | 7 | 2 | 5 | ㅡ |
| Other | 6 | 1 | 5 | ㅡ |
|  |  |  |  |  |
| T stage |  |  |  | **<0.01** |
| T1/T2 | 24 | 18 | 6 | ㅡ |
| T3/T4 | 15 | 1 | 14 | ㅡ |
|  |  |  |  |  |
| N stage |  |  |  | **<0.01** |
| N0 | 5 | 5 | 0 | ㅡ |
| N1/N2/N3 | 34 | 9 | 25 | ㅡ |
|  |  |  |  |  |
| Lymph node metastasis |  |  |  | **0.009** |
| N=0 | 17 | 13 | 4 | ㅡ |
| N≥1 | 22 | 8 | 14 | ㅡ |
|  |  |  |  |  |
