# Supplementary material for: MicroRNA-18a regulates the metastatic properties of oral squamous cell carcinoma cells via HIF-1α expression
Source: BMC Oral Health. 2022 Sep 5;22:378. doi: 10.1186/s12903-022-02425-6 (PMC9442921; doi:10.1186/s12903-022-02425-6)
Supplement: Supplementary file 2 — Additional file 2. Figure S1. The original picture of western blots of Figs. 2A were shown in Figs. S1 (the red box indicated the representative picture in duplicate). Protein expression of HIF-1α in YD-10B and HSC-2 cells under hypoxic conditions by western blot analysis. All membranes were cut into small pieces and incubated with each antibodies. GAPDH was used as the control gene. Merge images indicate combination with bright field and chemiluminescence. The chemiluminescence images were time exposures of minimum (Low exposure) and maximum (High exposure) duration by the Fusion Solo Vilber Lourmat system. All images were unprocessed files.Figure S2. The original picture of western blots of Figs. 3C were shown in Figs. S2 (the red box indicated the representative picture in duplicate). Protein expression of HIF-1α and PCNA induced by miRNA-18a mimics in YD-10B cells by western blot analysis. All membranes were cut into small pieces and incubated with each antibodies. GAPDH was used as the control gene. Merge images indicate combination with bright field and chemiluminescence. The chemiluminescence images were time exposures of minimum (Low exposure) and maximum (High exposure) duration by the Fusion Solo Vilber Lourmat system. All images were unprocessed files. Figure S3. The original picture of western blots of Figs. 3C were shown in Figs. S3 (the red box indicated the representative picture in duplicate). Protein expression of HIF-1α and PCNA induced by miRNA-18a mimics in HSC-2 cells by western blot analysis. All membranes were cut into small pieces and incubated with each antibodies. GAPDH was used as the control gene. Merge images indicate combination with bright field and chemiluminescence. The chemiluminescence images were time exposures of minimum (Low exposure) and maximum (High exposure) duration by the Fusion Solo Vilber Lourmat system. All images were unprocessed files. Figure S4. The original picture of western blots of Figs. 3D were shown in Figs. S4 [file 12903_2022_2425_MOESM2_ESM.docx]

The original picture of western blots was added as follows:


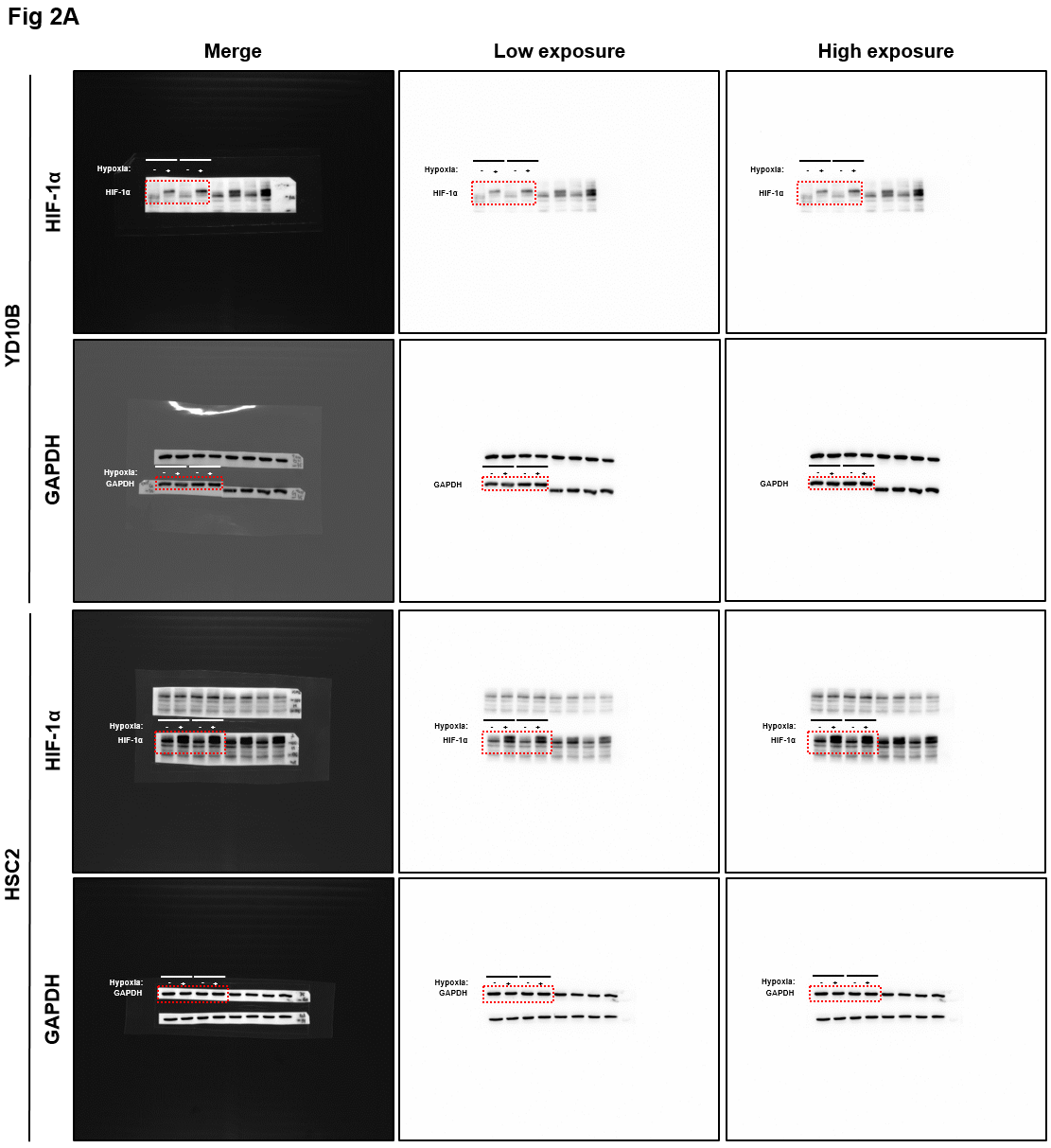


Figure S1. The original picture of western blots of Figs. 2A were shown in Figs. S1 (the red box indicated the representative picture in duplicate). Protein expression of HIF-1α in YD-10B and HSC-2 cells under hypoxic conditions by western blot analysis. All membranes were cut into small pieces and incubated with each antibodies. GAPDH was used as the control gene. Merge images indicate combination with bright field and chemiluminescence. The chemiluminescence images were time exposures of minimum (Low exposure) and maximum (High exposure) duration by the Fusion Solo Vilber Lourmat system. All images were unprocessed files.


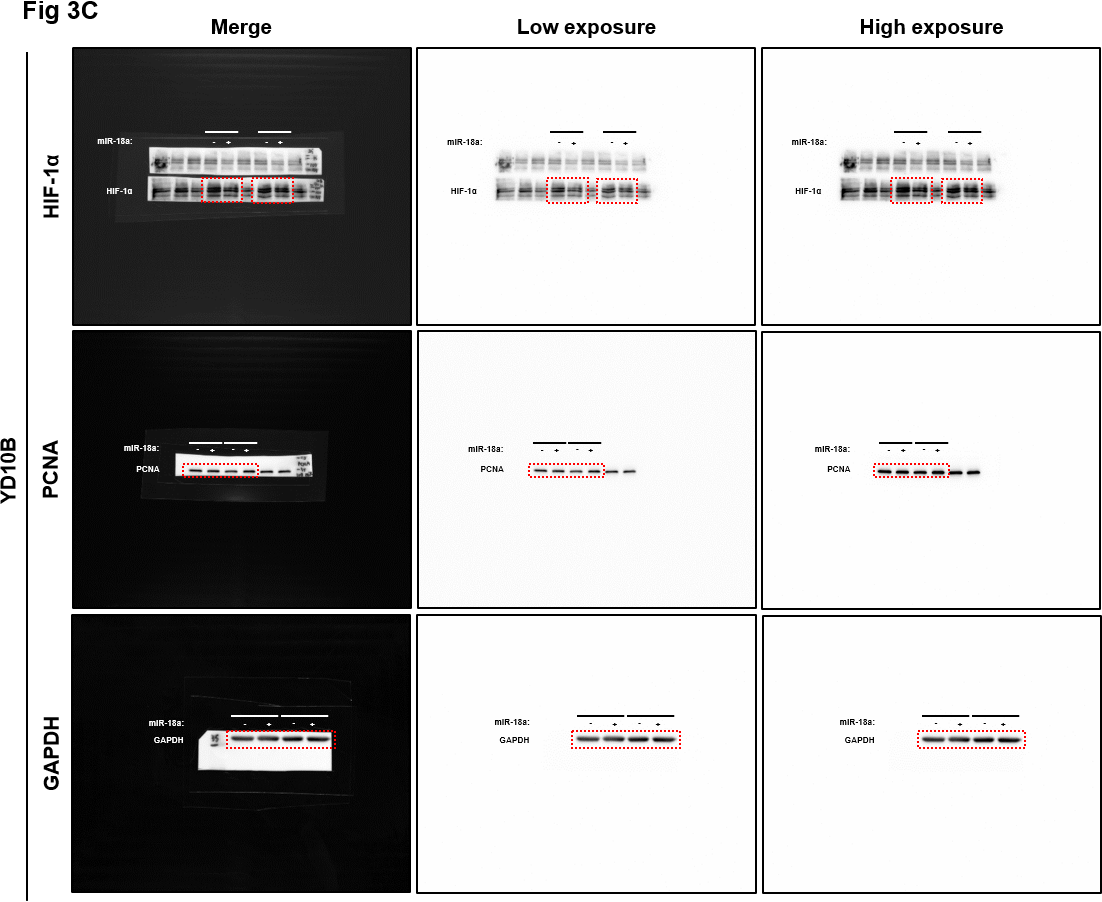


Figure S2. The original picture of western blots of Figs. 3C were shown in Figs. S2 (the red box indicated the representative picture in duplicate). Protein expression of HIF-1α and PCNA induced by miRNA-18a mimics in YD-10B cells by western blot analysis. All membranes were cut into small pieces and incubated with each antibodies. GAPDH was used as the control gene. Merge images indicate combination with bright field and chemiluminescence. The chemiluminescence images were time exposures of minimum (Low exposure) and maximum (High exposure) duration by the Fusion Solo Vilber Lourmat system. All images were unprocessed files.


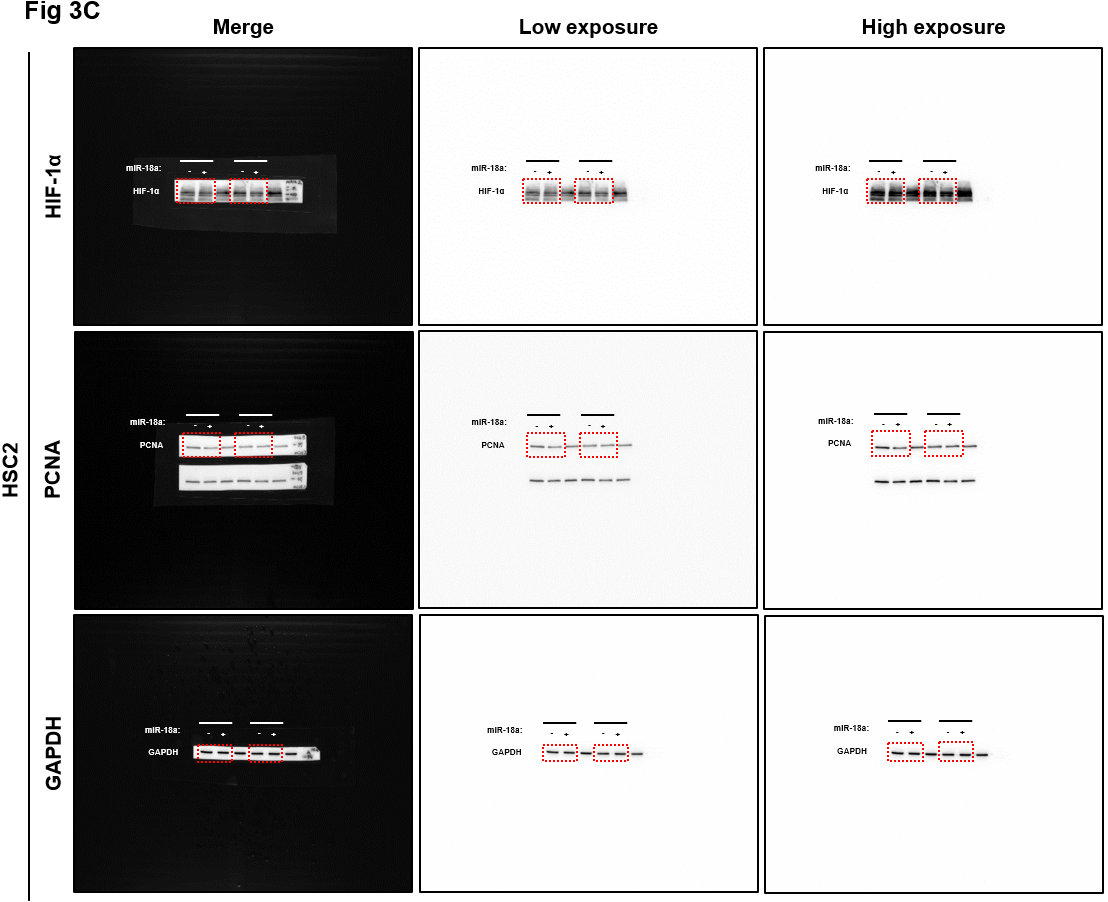


Figure S3. The original picture of western blots of Figs. 3C were shown in Figs. S3 (the red box indicated the representative picture in duplicate). Protein expression of HIF-1α and PCNA induced by miRNA-18a mimics in HSC-2 cells by western blot analysis. All membranes were cut into small pieces and incubated with each antibodies. GAPDH was used as the control gene. Merge images indicate combination with bright field and chemiluminescence. The chemiluminescence images were time exposures of minimum (Low exposure) and maximum (High exposure) duration by the Fusion Solo Vilber Lourmat system. All images were unprocessed files.


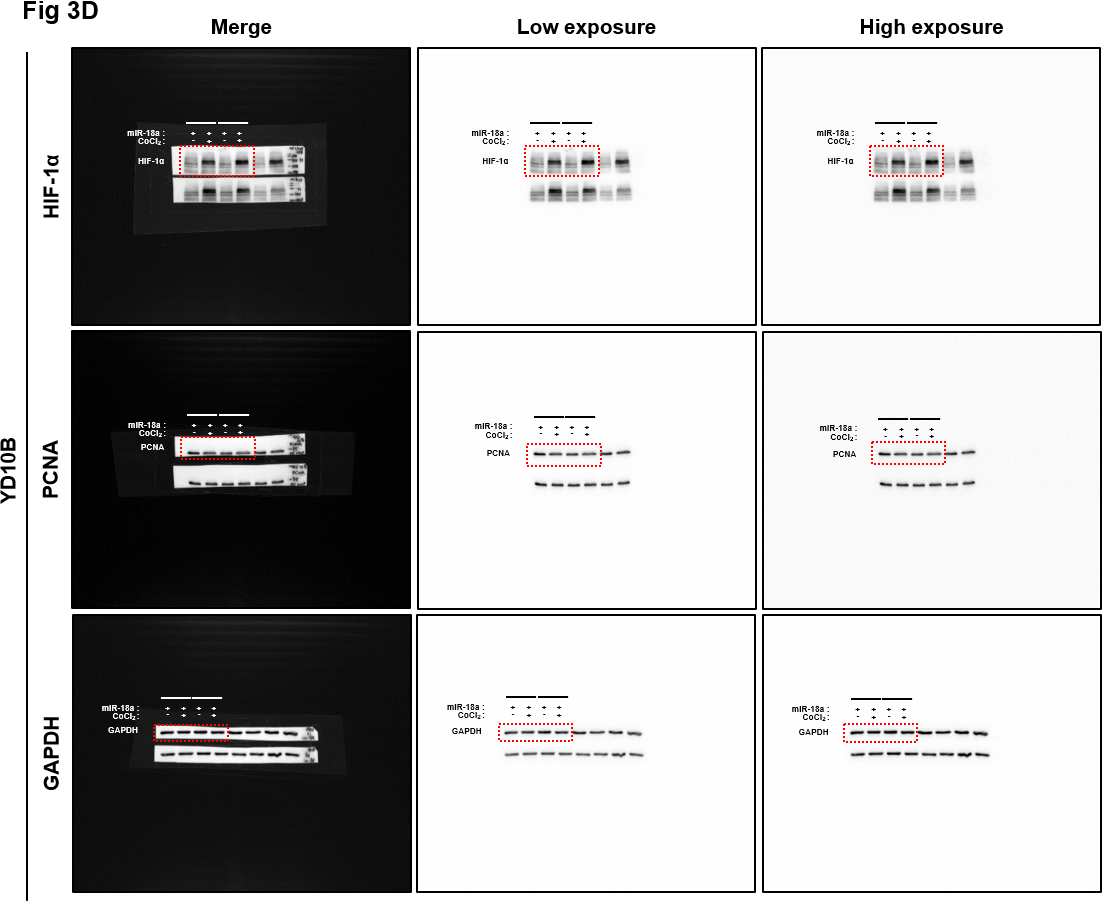


Figure S4. The original picture of western blots of Figs. 3D were shown in Figs. S4 (the red box indicated the representative picture in duplicate). Protein expression of HIF-1α and PCNA induced by miRNA-18a mimics or miRNA-18a and CoCl2 in YD-10B cells by western blot analysis. All membranes were cut into small pieces and incubated with each antibodies. GAPDH was used as the control gene. Merge images indicate combination with bright field and chemiluminescence. The chemiluminescence images were time exposures of minimum (Low exposure) and maximum (High exposure) duration by the Fusion Solo Vilber Lourmat system. All images were unprocessed files.


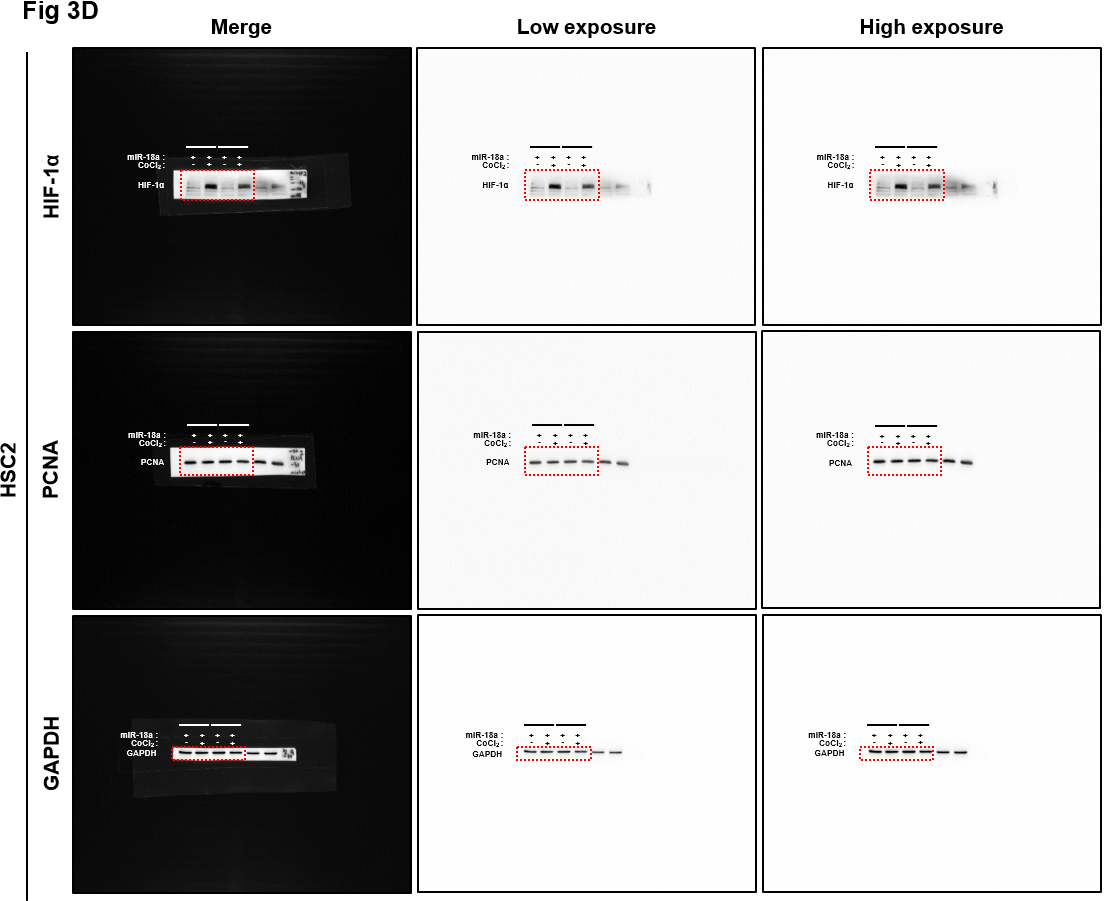


Figure S5. The original picture of western blots of Figs. 3D were shown in Figs. S5 (the red box indicated the representative picture in duplicate). Protein expression of HIF-1α and PCNA induced by miRNA-18a mimics or miRNA-18a and CoCl2 in HSC-2 cells by western blot analysis. All membranes were cut into small pieces and incubated with each antibodies. GAPDH was used as the control gene. Merge images indicate combination with bright field and chemiluminescence. The chemiluminescence images were time exposures of minimum (Low exposure) and maximum (High exposure) duration by the Fusion Solo Vilber Lourmat system. All images were unprocessed files.
